# Ethnic differences in grains consumption and their contribution to intake of B-vitamins: results of the Multiethnic Cohort Study

**DOI:** 10.1186/1475-2891-12-65

**Published:** 2013-05-20

**Authors:** Sangita Sharma, Tony Sheehy, Laurence N Kolonel

**Affiliations:** 1 Department of Medicine, Aboriginal and Global Health Research Group, University of Alberta, 5-10 University Terrace, 8303 112 Street, Edmonton, Alberta T6G 2T4, Canada; 2 Department of Food and Nutritional Sciences, University College Cork, Cork, Republic of Ireland; 3 Epidemiology Program, Cancer Research Center of Hawaii, University of Hawaii, 1236 Lauhala Street, Honolulu, Hawaii 96813, USA

**Keywords:** Whole grains, Refined grains, B vitamins, Ethnicity

## Abstract

**Background:**

Research indicates that a diet rich in whole grains may reduce the risk of prevalent chronic diseases, including cardiovascular disease, diabetes, and some cancers, and that risk for these diseases varies by ethnicity. The objective of the current study was to identify major dietary sources of grains and describe their contribution to B vitamins in five ethnic groups.

**Methods:**

A cross-sectional mail survey was used to collect data from participants in the Multiethnic Cohort Study in Hawaii and Los Angeles County, United States, from 1993 to 1996. Dietary intake data collected using a quantitative food frequency questionnaire was available for 186,916 participants representing five ethnic groups (African American, Latino, Japanese American, Native Hawaiian and Caucasian) aged 45–75 years. The top sources of grain foods were determined, and their contribution to thiamin, riboflavin, niacin, vitamin B6, and folic acid intakes were analyzed.

**Results:**

The top source of whole grains was whole wheat/rye bread for all ethnic-sex groups, followed by popcorn and cooked cereals, except for Native Hawaiian men and Japanese Americans, for whom brown/wild rice was the second top source; major contributors of refined grains were white rice and white bread, except for Latinos. Refined grain foods contributed more to grain consumption (27.1-55.6%) than whole grain foods (7.4-30.8%) among all ethnic-sex groups, except African American women. Grain foods made an important contribution to the intakes of thiamin (30.2-45.9%), riboflavin (23.1-29.2%), niacin (27.1-35.8%), vitamin B6 (22.9-27.5%), and folic acid (23.3-27.7%).

**Conclusions:**

This is the first study to document consumption of different grain sources and their contribution to B vitamins in five ethnic groups in the U.S. Findings can be used to assess unhealthful food choices, to guide dietary recommendations, and to help reduce risk of chronic diseases in these populations.

## Background

Recent research has shown that a diet rich in whole grains may reduce the risk of certain diseases, such as cardiovascular disease (CVD) [1,2], diabetes [3], obesity [4], and certain cancers [3,5]. The U.S. Dietary Guidelines recommend consumption of three or more ounce-equivalents of whole grain products per day to reduce the risk of several chronic diseases and to help with weight maintenance [6]. Evidence is conflicting about associations between refined grain consumption and CVD [7] and anthropometric indices [8]; however, there is some evidence that consumption of refined grains is associated with cancer risk [9,10].

Whole grains contain many bioactive components that might be responsible for their protective effect, including dietary fiber and resistant starch, as well as vitamins, minerals, and phytoestrogens [11-13]. Fiber consumption is related to decreased risk of cancer, CVD, diabetes, and obesity [14-16]. B vitamins in grains are water-soluble and are necessary to support and increase the rate of metabolism, maintain healthy skin and muscle tone, enhance immune and nervous system function, and help prevent anemia [17,18]. Folic acid, vitamins B6, and B12 reduce the risk of CVD and cancer [14,19]. In addition, folic acid fortification of grains is associated with reduced incidence of neural tube and other birth defects [20].

Despite a growing awareness of the benefits of whole grains, their consumption remains inadequate in the U.S. population. Data from the 1994–1996 Continuing Survey of Food Intakes by Individuals (CSFII) indicated that the mean daily intake of foods containing whole grains was just one serving per day [21]. These CSFII dietary data also showed that only 7% of Americans ate the recommended three or more servings of whole grain foods per day [22]. A more recent report of the National Health and Nutrition Examination Survey (NHANES) 1999–2002, indicated that only 4% of individuals aged 12 years and older met the whole grain recommendation [23]. In addition, most grain products consumed in the U.S. are highly refined and contain more starch but less fiber, vitamins, and minerals [24].

Chronic diseases, including cancer, CVD, and diabetes, are the leading causes of death for men and women of all races and ethnicities in the U.S. However, risks vary by ethnic group [25]. For example, age-adjusted cancer mortality rates in 2008 for men and women, respectively, were 322 and 189 per 100,000 for African Americans, 235 and 161 for Caucasians, 142 and 97 for Asians, and 162 and 107 for Latinos [26]. Consumption of whole grain products could become a key component in the reduction of chronic diseases in these populations [27]. Grains can also be significant sources of B vitamins [28], which may play an important role in chronic disease prevention [29], including conditions such as cognitive decline and dementia [30]. With the aging population in countries such as the US [31], the implementation of cost-effective preventive measures such as diet is becoming increasingly important. In addition, grain consumption varies by race and ethnicity [24]. Information regarding ethnic differences in dietary sources of grain and their contribution to B vitamins can potentially be used to tailor nutrition education and intervention programs for specific ethnic groups. However, to our knowledge, no studies have provided information regarding grain foods commonly consumed among different ethnic groups in the U.S. using a standardized dietary assessment method. The objective of the present study was to describe dietary sources of grains and their contribution to thiamin, riboflavin, niacin, vitamin B6, and folic acid in five ethnic groups in the U.S.

## Methods

The Multiethnic Cohort (MEC) was established in1993 through 1996 to examine lifestyle risk factors, especially diet, in relation to cancer risk among men and women of five different ethnic groups in Hawaii and Los Angeles, California [32]. Subjects completed a 26-page, self-administered mailed questionnaire which included a quantitative food frequency questionnaire (QFFQ) with 180 food items. Details on the development and validation of this QFFQ are reported elsewhere [32,33]. Briefly, it was developed specifically for the study population based on 3-day measured food records from approximately 60 men and 60 women from each ethnic group. The QFFQ included ethnic-specific foods irrespective of their contribution to daily nutrient intake [32]. Acceptable correspondence between the questionnaire and multiple 24-hour recalls for each ethnic-sex group was shown in a calibration sub-study [33]. Average correlation coefficients for nutrients (protein, fats, carbohydrates, calcium, vitamins A and C, beta-carotene and dietary fibre) ranged from 0.26 in African American women to 0.57 in Caucasian men, while for nutrient densities, average correlations ranged from 0.57 to 0.74 across ethnic-sex groups [33]. Although correlations coefficients for specific B vitamins were not reported, these results suggest that the QFFQ is an adequate instrument for assessment of nutrient intake. The study protocol was approved by the Institutional Review Boards of the University of Hawaii and the University of Southern California.

Of the 215,251 participants, 16% were African American (AfAm), 22% Latino, 26% Japanese American (JpAM), 7% Native Hawaiian (NH), 23% Caucasian, and 6% others. Response rates varied from 20% in Latinos to 49% in JpAm. Participants with extreme energy and macronutrient values (mean +/− 3 SDs for energy and mean +/− 3.5 SDs for fats, proteins, and carbohydrates) were excluded. Likewise, individuals reporting a mixed ethnic background and Latinos born in the Caribbean, due to their small number, were not included in our analysis. In addition, Latinos born in Mexico and Central/South America (Latino-Mexico) and Latinos born in the U.S. (Latino-US) were separated because food consumption patterns differ substantially between Latinos by birthplace [34]. Therefore, a total of 189,916 remained in the analysis of the present study, including; 31,852 AfAm, 13,629 NH, 51,248 JpAm, 42,951 Latinos (21,083 Latino-Mexico and 21,868 Latino-US), and 47,236 Caucasians.

The detailed methods for developing and calculating food group servings for the MEC have been described previously [33]. Based on servings specified in the Food Guide Pyramid, an individual’s serving for each food group was computed by summing the daily servings across the appropriate food items on the QFFQ. Average daily Food Guide Pyramid servings in the MEC have been reported previously [34,35]. Nutrient intakes were analyzed based on a food composition table developed specifically for this multiethnic population, which was extended and adapted from the USDA food composition database [36].

In this study, the top food sources of whole and refined grains as well as total grain foods were identified. Similar foods were combined to calculate the percent contributions of foods, including grain products, to daily intakes of B vitamins (thiamin, riboflavin, niacin, vitamin B6, and folic acid). The top food sources of vitamin B12 have been reported elsewhere [37].

## Results

The mean ages of participants ranged from 56 to 62 years between ethnic-sex groups (data not shown). Among the five ethnic groups, JpAm women had the lowest mean BMI at 23.1 kg/m^2^ and NH men had the highest mean BMI with 28.5 kg/m^2^. NH also had the highest energy intake among men (2,760 kcal ± 1,311) and women (2,370 kcal ± 1,263).

### Sources of grain intake

Table 1 lists the top five sources of whole grains by ethnic-sex group. Whole wheat/rye bread was the most commonly reported whole grain food for all ethnic-sex groups (25.2-37.5%). The three major commonly consumed whole grain foods were whole wheat/rye bread, popcorn, and cooked cereals (the top three combined contributed between 50.6-68.7%), except for NH men, JpAm and Caucasians for whom brown/wild rice or other breads followed whole wheat/rye bread. In contrast, highly fortified cereals contributed only 2.4-5.4% to whole grain intake (not included in the top five, thus data not shown).

**Table 1:** Five major sources of whole grains and their percent contribution by ethnic group and gender*

	**African Americans**		**Native Hawaiians**		**Japanese Americans**		**Latino-Mexico**		**Latino-US**		**Caucasians**	
**Food items**	**Women (%)**	**Men (%)**	**Women (%)**	**Men (%)**	**Women (%)**	**Men (%)**	**Women (%)**	**Men(%)**	**Women (%)**	**Men (%)**	**Women (%)**	**Men (%)**
Whole wheat or rye bread	**32.9**	**37.5**	**31.7**	**32.5**	**28.9**	**28.7**	**28.0**	**25.2**	**28.0**	**28.0**	**28.3**	**28.1**
Popcorn	23.7	16.3	14.4	10.8	8.6	7.8	9.1	9.1	17.7	15.8	17.8	17.5
Cooked cereals	12.1	11.3	10.1	7.7	11.8	10.1	17.9	16.3	14.2	12.0	8.4	6.9
Bran or high fiber cereals	6.4	7.0	n/a	n/a	n/a	7.3	8.2	7.5	6.9	7.5	7.8	8.7
Other bread	5.7	5.3	8.8	8.2	8.3	n/a	6.9	n/a	7.0	n/a	10.8	8.8
Brown or wild rice	n/a	n/a	8.2	11.3	16.7	17.5	n/a	n/a	n/a	n/a	n/a	n/a
White bread	n/a	n/a	n/a	n/a	n/a	n/a	n/a	6.8	n/a	n/a	n/a	n/a
Potato/Corn/Tortilla	n/a	n/a	n/a	n/a	n/a	n/a	n/a	n/a	n/a	6.3	n/a	n/a
**Total [%]**	**80.8**	**77.4**	**73.2**	**70.5**	**74.3**	**71.4**	**70.1**	**64.9**	**73.8**	**69.6**	**73.1**	**70.0**

* Top source for each ethnic-sex group is bolded; items that did not appear in the top 5 for a particular ethnic sex group are indicated by n/a (not applicable).

White rice was the most commonly consumed refined grain food item [10.3-54.1%], followed by white bread (5.6-10.4%) among all ethnic groups, except for Latinos who primarily consumed Latino corn (tortilla/bread) and rolls (buns/biscuits) (Table 2). However, the contribution of white rice was much higher in NH and JpAm (36.7-54.1%) compared to other ethnic groups (10.3-17.1%). Mexican or Spanish rice appeared among the top five sources for Latino-Mexico only, while pizza and pancakes/French toast each appeared only once among the top five (for NH men and Caucasian men, respectively).

**Table 2:** Five major sources of refined grains and their percent contribution by ethnic group and gender*

	**African Americans**		**Native Hawaiians**		**Japanese Americans**		**Latino-Mexico**		**Latino-US**		**Caucasians**	
**Food items**	**Women (%)**	**Men (%)**	**Women (%)**	**Men (%)**	**Women (%)**	**Men (%)**	**Women (%)**	**Men (%)**	**Women (%)**	**Men (%)**	**Women (%)**	**Men (%)**
White rice	**10.3**	**11.8**	**36.7**	**46.4**	**47.0**	**54.1**	**7.1**	**5.7**	**8.5**	**8.1**	**12.8**	**17.1**
White bread	8.4	10.4	7.6	7.2	5.9	5.6	7.1	7.3	8.7	9.1	9.3	9.3
Rolls/Buns/Biscuits	6.4	6.1	4.3	3.1	3.9	2.5	7.8	8.3	14.9	14.4	9.2	6.6
Corn tortilla/bread	6.4	5.3	n/a	n/a	n/a	n/a	27.8	28.7	10.4	9.5	n/a	n/a
Sweet rolls / Coffee cake	n/a	4.3	n/a	n/a	n/a	n/a	n/a	n/a	3.5	4.2	n/a	n/a
Ramen or saimin	n/a	n/a	3.2	2.5	3.0	2.8	n/a	n/a	n/a	n/a	n/a	n/a
Crackers and pretzels	n/a	n/a	2.9	n/a	3.2	2.0	n/a	n/a	n/a	n/a	6.2	n/a
Mexican or Spanish rice	n/a	n/a	n/a	n/a	n/a	n/a	4.0	3.8	n/a	n/a	n/a	n/a
Spaghetti / Ravioli / Lasagna	4.1	n/a	n/a	n/a	n/a	n/a	n/a	n/a	n/a	n/a	6.0	5.5
Pizza	n/a	n/a	n/a	n/a	n/a	n/a	n/a	n/a	n/a	n/a	n/a	4.8
Pancakes / French toast	n/a	n/a	n/a	2.3	n/a	n/a	n/a	n/a	n/a	n/a	n/a	n/a
**Total [%]**	**35.6**	**37.9**	**54.7**	**61.5**	**63.0**	**67.0**	**53.8**	**53.8**	**46.0**	**45.3**	**43.5**	**43.3**

* Top source for each ethnic-sex group is bolded; items that did not appear in the top 5 for a particular ethnic sex group are indicated by n/a (not applicable).

The contribution of the top ten grain products to total grain intake (data not shown) varied between 56.6% (Latino-US men) and 71.6% (JpAm men). Refined grain foods contributed the most to grain consumption (27.1-55.6%), followed by whole grain foods (7.4-30.8%) among all ethnic-sex groups, except for AfAm women who primarily consumed whole grain foods. White rice was the top source of grain foods among JpAm, NH, and Caucasian men (12.0-44.1%), while AfAm and Caucasians women primarily consumed whole wheat/rye bread (10.5-13.7%). Corn (tortilla/bread) was the top contributor only for Latino-Mexico and rolls (buns/biscuits) for Latino-US.

### Contribution of grain to B vitamins

Grain foods contributed substantially to thiamin intake among all ethnic-sex groups (Table 3). The two main thiamin sources were cereals (9.7-22.3%) and bread (8.2-16.9%) for all ethnic-sex groups, with the exception of JpAm men, where rice was the secondary contributor (Table 3). Rice was also among the top five contributors for all ethnic-sex groups (3.4-13.7%), except African American women and Caucasians of both sexes, for whom pasta with tomato or cheese was among the top five instead. In addition to grain foods, orange/grapefruit/pomelos were among the top five contributors for all ethnic-sex groups (4.9-6.9%), and pork/ham for all but Latino-Mexican men.

**Table 3:** Five major sources of thiamin and their percent contribution by ethnic group and gender*

	**African Americans**		**Native Hawaiians**		**Japanese Americans**		**Latino-Mexico**		**Latinos-US**		**Caucasians**	
**Food items**	**Women (%)**	**Men (%)**	**Women (%)**	**Men (%)**	**Women (%)**	**Men (%)**	**Women (%)**	**Men (%)**	**Women (%)**	**Men (%)**	**Women (%)**	**Men (%)**
Cereals	**19.2**	**18.4**	**15.6**	**13.9**	**15.6**	**14.8**	**22.3**	9.7	**17.1**	**15.6**	**18.8**	**19.0**
Bread	11.2	11.8	11.6	11.2	11.4	10.1	8.2	**16.9**	12.8	11.9	13.8	12.3
Orange/GF/pomelo	6.1	6.7	5.6	4.9	6.9	5.3	6.2	4.9	5.6	4.9	5.4	4.9
Pork & ham	3.4	4.9	4.4	5.6	3.2	4.5	4.2	n/a	3.9	5.1	3.1	4.4
Rice	n/a	4.6	6.4	9.3	10.5	13.7	3.4	5.1	6.5	8.7	n/a	n/a
Pasta with TS or cheese	4.3	n/a	n/a	n/a	n/a	n/a	n/a	n/a	n/a	n/a	5.1	5.0
Muffins/doughnuts	n/a	n/a	n/a	n/a	n/a	n/a	n/a	4.5	n/a	n/a	n/a	n/a
**Total [%]**	**44.2**	**46.4**	**43.6**	**44.9**	**47.6**	**48.4**	**44.3**	**41.1**	**45.9**	**46.2**	**46.2**	**45.6**

* Top source for each ethnic-sex group is bolded; items that did not appear in the top 5 for a particular ethnic sex group are indicated by n/a (not applicable); *TS*, Tomato sauce; *GF*, Grapefruits.

Grain foods contributed considerably to the top five food sources of riboflavin intake (21.1-28.5%), followed by milk and dairy products (3.2-10.7%) among all ethnic groups (Table 4). Among the top five dietary sources, the two major contributors of riboflavin for all ethnic-sex groups were cereals (5.0-17.4%) and bread (7.1-19.4%). Low fat milk was the third highest contributor for all but NH and JpAm men. Surprisingly, beer appeared in the top five for several male groups (NH, JpAm, Latino-US and Caucasians).

**Table 4:** Five major sources of riboflavin and their percent contribution by ethnic group and gender*

	**African Americans**		**Native Hawaiians**		**Japanese Americans**		**Latino-Mexico**		**Latino-US**		**Caucasians**	
**Food items**	**Women (%)**	**Men (%)**	**Women (%)**	**Men (%)**	**Women (%)**	**Men (%)**	**Women (%)**	**Men (%)**	**Women (%)**	**Men (%)**	**Women (%)**	**Men (%)**
Cereals	**17.4**	**17.2**	**14.0**	**12.8**	**14.4**	**14.0**	5.0	8.6	**15.4**	**14.4**	**16.2**	**16.8**
Bread	7.1	7.7	7.7	7.4	8.0	7.1	**19.4**	**10.8**	8.4	7.8	8.5	7.6
Low fat milk	4.8	4.2	4.9	4.1	3.9	3.2	6.4	5.5	4.6	4.1	5.3	5.2
Chicken/turkeys	3.3	3.5	n/a	n/a	n/a	4.2	n/a	n/a	n/a	n/a	n/a	n/a
Pasta with TS or cheese	3.7	3.2	n/a	2.8	n/a	n/a	n/a	3.2	3.4	3.2	4.2	4.1
Beer	n/a	n/a	n/a	4.5	n/a	4.9	n/a	n/a	n/a	4.1	n/a	3.7
Eggs	n/a	n/a	n/a	3.6	n/a	n/a	n/a	n/a	n/a	n/a	n/a	n/a
Nonfat milk	n/a	n/a	3.2	n/a	5.1	n/a	4.3	n/a	3.1	n/a	5.2	n/a
Muffins/doughnuts	n/a	n/a	n/a	n/a	n/a	n/a	n/a	3.4	n/a	n/a	n/a	n/a
Rice	n/a	n/a	n/a	n/a	3.3	n/a	n/a	n/a	n/a	n/a	n/a	n/a
Bananas	n/a	n/a	n/a	n/a	n/a	n/a	3.4	n/a	n/a	n/a	n/a	n/a
**Total [%]**	**36.3**	**35.8**	**29.8**	**35.2**	**34.7**	**33.4**	**38.5**	**31.5**	**34.9**	**33.6**	**39.4**	**37.4**

* Top source for each ethnic-sex group is bolded; items that did not appear in the top 5 for a particular ethnic sex group are indicated by n/a (not applicable); *TS*, Tomato sauce.

Several grain dishes (cereals, bread, rice and pasta) were also among the top five contributors to niacin intake (Table 5). The combined contribution of these four dishes to niacin intake among the different ethnic-sex groups was between 20.8 and 32.7%. Cereals were the top dietary source of niacin (11.8% in NH men to 20.3% in Latino-Mexican women) for all but Latino-Mexican men, for whom bread was the top contributor (11.9%). Other significant contributors among all ethnic-sex groups were chicken/turkey (8.3-12.6%) and fish (3.4-8.1%) dishes. Similar to the findings for riboflavin sources, beer appeared again on the top five contributor list, but only for Caucasian men.

**Table 5:** Five major sources of niacin and their percent contribution by ethnic group and gender

	**African Americans**		**Native Hawaiians**		**Japanese Americans**		**Latino-Mexico**		**Latino-US**		**Caucasians**	
**Food items**	**Women (%)**	**Men (%)**	**Women (%)**	**Men (%)**	**Women (%)**	**Men (%)**	**Women (%)**	**Men (%)**	**Women (%)**	**Men (%)**	**Women (%)**	**Men (%)**
Cereals	**17.7**	**16.5**	**13.9**	**11.8**	**13.5**	**12.0**	**20.3**	8.9	**15.3**	**13.5**	**17.6**	**17.0**
Chicken/turkey	12.6	12.1	8.6	8.3	9.1	9.1	10.7	8.3	9.7	9.4	9.9	8.9
Bread	8.1	8.3	8.2	7.3	8.2	6.5	6.1	**11.9**	9.1	7.8	10.1	8.4
Fish	5.7	5.2	7.7	8.1	7.5	8.2	3.4	5.5	5.9	6.1	5.4	5.5
Rice	n/a	4.3	6.0	8.0	10.0	11.6	3.1	n/a	6.0	7.5	n/a	n/a
Pasta with TS or cheese	3.9	n/a	n/a	n/a	n/a	n/a	n/a	n/a	n/a	n/a	5.0	n/a
Taco salad	n/a	n/a	n/a	n/a	n/a	n/a	n/a	5.5	n/a	n/a	n/a	n/a
Beer	n/a	n/a	n/a	n/a	n/a	n/a	n/a	n/a	n/a	n/a	n/a	4.7
**Totals [%]**	**48.0**	**46.4**	**44.4**	**43.5**	**48.3**	**47.4**	**43.6**	**40.1**	**46.0**	**44.3**	**48.0**	**44.5**

* Top source for each ethnic-sex group is bolded; items that did not appear in the top 5 for a particular ethnic sex group are indicated by n/a (not applicable); *TS*, Tomato sauce.

As seen in Table 6, grain foods also contributed substantially to vitamin B6 intake. The combined contribution of cereals, rice and bread to vitamin B6 intake ranged from 18.1% among Caucasian men, up to 25.0% among JpAm men. Cereals alone accounted for between 9.7 and 18.1% of niacin intake and were the top contributor for all but JpAm women and Latino-Mexican men. Bananas were also a considerable source of vitamin B6 among all groups (9.0-16.0%), and were the highest contributor for JpAm women and Latino-Mexican men. Beer was a top five contributor again, for all male groups except Latino-Mexican men.

**Table 6:** Five major sources of vitamin B6 and their percent contribution by ethnic group and gender*

	**African Americans**		**Native Hawaiians**		**Japanese Americans**		**Latino-Mexico**		**Latino-US**		**Caucasians**	
**Food items**	**Women (%)**	**Men (%)**	**Women (%)**	**Men (%)**	**Women (%)**	**Men (%)**	**Women (%)**	**Men (%)**	**Women (%)**	**Men (%)**	**Women (%)**	**Men (%)**
Cereals	**17.9**	**17.6**	**14.2**	**12.9**	13.7	**13.1**	**19.0**	9.7	**15.7**	**14.8**	**17.6**	**18.1**
Bananas	11.9	11.1	11.3	9.0	**14.3**	10.9	16.0	**14.1**	13.3	10.1	13.2	11.0
Chicken/turkeys	6.7	6.7	4.6	4.7	4.8	5.2	5.3	4.8	5.2	5.4	5.1	4.9
Rice	n/a	4.3	5.7	8.1	9.5	11.9	n/a	4.4	5.8	7.6	n/a	n/a
Beer	n/a	3.5	n/a	5.5	n/a	5.3	n/a	n/a	n/a	5.1	n/a	5.2
Bread	3.2	n/a	n/a	n/a	n/a	n/a	2.8	4.9	3.5	n/a	3.9	n/a
Potatoes	n/a	n/a	n/a	n/a	n/a	n/a	n/a	n/a	n/a	n/a	3.6	3.7
Orange/GF/pomelo	3.0	n/a	n/a	n/a	n/a	n/a	2.9	n/a	n/a	n/a	n/a	n/a
Stir-fried MT & Veg	n/a	n/a	n/a	n/a	3.6	n/a	n/a	n/a	n/a	n/a	n/a	n/a
Taro	n/a	n/a	3.8	n/a	n/a	n/a	n/a	n/a	n/a	n/a	n/a	n/a
**Total [%]**	**42.7**	**43.2**	**39.6**	**40.2**	**45.9**	**46.4**	**46.4**	**37.9**	**43.5**	**43.0**	**43.4**	**42.9**

* Top source for each ethnic-sex group is bolded; items that did not appear in the top 5 for a particular ethnic sex group are indicated by n/a (not applicable); *GF*, Grapefruits; *Veg*, vegetables; *MT*, meat.

Among the top five contributors for folic acid, cereals and bread accounted for between 19.9% (among Latino-Mexican men) and 27.7% (among Caucasian men) of nutrient intake (Table 7). Among all ethnic groups, the top contributor to folic acid intake was cereals (11.9-22.3%), followed by orange/grapefruit/pomelo (8.9-12.7%). Bread was also among the top five major sources of folic acid intake for all groups, except for Latino-Mexico women. Again, beer appeared among the top five in men only, and contributed to 4.5% of folate intake for NH men, and 4.3% for JpAm men.

**Table 7:** Five major sources of folic acid and their percent contribution by ethnic group and gender*

	**African Americans**		**Native Hawaiians**		**Japanese Americans**		**Latino-Mexico**		**Latinos-US**		**Caucasians**	
**Food items**	**Women (%)**	**Men (%)**	**Women (%)**	**Men (%)**	**Women (%)**	**Men (%)**	**Women (%)**	**Men (%)**	**Women (%)**	**Men (%)**	**Women (%)**	**Men (%)**
Cereals	**20.3**	**20.9**	**17.7**	**17.3**	**16.4**	**17.4**	**22.3**	**11.9**	**18.6**	**19.0**	**20.0**	**21.8**
Orange/GF/pomelo	10.4	12.7	10.4	9.7	12.0	10.2	8.9	9.5	10.3	9.5	9.7	9.5
Bread	5.0	5.9	5.8	5.9	5.5	5.3	n/a	8.0	6.0	5.9	6.2	5.9
Beans	5.4	5.5	n/a	n/a	n/a	n/a	8.2	8.0	4.6	4.9	3.9	4.0
Dark greens	5.6	3.7	6.5	5.1	7.6	6.2	3.6	3.3	6.0	4.4	6.3	4.3
Beer	n/a	n/a	n/a	4.5	n/a	4.3	n/a	n/a	n/a	n/a	n/a	n/a
Tropical fruits	n/a	n/a	3.7	n/a	5.5	n/a	4.7	n/a	n/a	n/a	n/a	n/a
**Total [%]**	**46.7**	**48.7**	**44.1**	**42.5**	**47.0**	**43.4**	**47.7**	**40.7**	**45.5**	**43.7**	**46.1**	**45.5**

* Top source for each ethnic-sex group is bolded; items that did not appear in the top 5 for a particular ethnic sex group are indicated by n/a (not applicable). GF=Grapefruits.

## Discussion

These findings reveal sex and ethnic differences in grain foods consumption. In particular, JpAm men had the highest contribution of refined grain food, while AfAm women had the highest contribution of whole grain food to total grain intake. Ethnic differences in the consumption of grain foods have been previously reported [38,39]. For example, Champagne et al. [38] found that cereal consumption was lower in European Americans when compared with AfAm. Differences in grain consumption by sex have also been previously described by Maras et al. [40]; women consumed more whole grain and less refined grain products than men. In contrast, AfAm women have the highest rates of overweight/obesity in the US [25]. Thus, it is important to evaluate ethnic-sex differences in food group consumption in light of the current obesity epidemic in the United States.

Major sources of whole grains in this study were whole wheat/rye bread, popcorn, cooked cereals, and wild rice. In the Baltimore Longitudinal Study of Aging, including 1,516 men and women (aged 62.1 ± 16.0 years), Maras et al. [40] found that top contributors of whole grains were ready-to-eat breakfast cereals, hot breakfast cereals, multi-grain bread, and whole wheat bread. A recent report of the 2001–2002 NHANES indicated that ready-to-eat cereals and hot cereals contributed more than 40% to whole grain consumption, followed by yeast breads, popcorn, and crackers [41]. In our study, major contributors of refined grains were white rice and white bread, except for Latinos who primarily consumed Latino corn/tortilla/bread and rolls/buns/biscuits. Data from the 2001–2002 NHANES indicated that yeast breads contributed 26% to non-whole grains followed by grain-based desserts, pizza, pasta and pasta dishes, and Mexican dishes [41]. However, most previous studies included mainly Caucasian populations and discrepancy between results could also be due to different methods, and geographical and cultural influences [42,43].

Results of the present study also indicate that the contribution of refined grain foods exceeded that of whole grain products among all ethnic/racial groups, except for AfAm women who primarily consumed whole grain foods. Such trends were previously observed in the US [7,44], the UK [45], and Mediterranean countries [46]. Factors responsible for these trends include confusion about which foods contain whole grains as well as the assumption that whole grains are darker in color than refined grains even though color additives are often used to change the color of foods [40]. Furthermore, many consumers associate whole grains with the coarse textures and flavors found in oat bran, wheat germ, and whole grain flours [40]. According to Baker et al. [42], consumers may not purchase whole grain foods due to higher cost. Finally, it is important to note that although many refined grain products are enriched with niacin, riboflavin, thiamin, and iron, enrichment doesn’t restore insoluble fiber and other nutrients that are lost during the milling process [47]. Our results underscore the need to encourage the consumption of whole grains instead of refined grains among ethnic/racial groups to reduce risk of chronic diseases.

Grain foods made an important contribution to the mean daily intakes of thiamin, riboflavin, niacin, pyridoxine, and folic acid. Previous studies have also demonstrated a positive impact of grain foods on intake of B vitamins [48,49]. Breakfast cereal consumption, for example, was associated with an adequate level of micronutrient intake [50]. Furthermore, some studies have shown that breakfast cereal consumers had higher intakes of thiamin, riboflavin, and folic acid compared with non-consumers [50,51]. The high level of vitamin B fortification in many breakfast cereals may explain the high contribution of these foods to vitamin B intake observed in this study [52]. Conversely, intake of refined grains, white rice and breads in particular, was relatively high among all ethnic-sex groups in this study, but the contribution of these foods to B vitamins was comparatively low. These findings are not surprising, given that the level of vitamin B fortification for breakfast cereals tends to be higher than that for rice [52,53]. Additional fortification of refined grains which make a considerable contribution to this food group intake may present an opportunity to address nutritional deficiencies in some populations.

Intakes of thiamin, riboflavin, and niacin have been reported to be low among older men and women (60–80 years) in the lipid research clinics program prevalence study [54]. In 2003–2006, 13% of non-Hispanic white women as well as Mexican American women had inadequate total folic acid intakes [55]. Another previous study found that 13% of AfAm, 6% of Caucasian, and 3% of Hispanic women reported inadequate intakes of vitamin B6 [56]. Results from this paper may potentially be used to help ease some of these concerns through the development of food-based ethnic-specific dietary guidelines. For example, recommendations based on increased consumption of whole wheat/rye bread could help increase thiamin, riboflavin, niacin, and vitamin B6 intakes, and would result in a diet with better nutrient quality. Results of this study underscore the need for both individualized nutrition interventions as well as environmental interventions to influence consumption patterns of these five ethnic groups.

Limitations of this study include possible recall bias. Also, measurement error is known to be higher with FFQs compared to other methods [57]. Unfortunately, the methodology used to validate the accuracy of the QFFQ used in this study to capture nutrient intake did not assess B vitamins [33]. While this limitation is a concern, the results from that study indicate that the tool is a valid instrument for assessment of several nutrients, and thus the findings from that study do support the application to a wider range of nutrients. The major strength of this paper lies in its large sample size and the use of a QFFQ developed and validated specifically for the multiethnic group to assure standardized data collection among the five ethnic/racial groups. A standardized food grouping methodology for grains and their subgroups was used and based on the USDA food groupings. Further, a comparison of the cohort distributions by educational levels and marital status with corresponding census data for the two geographic areas confirmed that the cohort was representative of the general population. However, the data available for this analyses were collected over 15 year ago, thus it is possible that changes in dietary sources or consumption patterns have occurred over time, which may have impacted the generalisability to current populations. Analyses of more recent data would be useful to clarify and confirm the finding from this study.

## Conclusions

The present study indicates ethnic-sex differences in grain food consumption and in their contributions to B vitamin intakes. Refined grain products contributed more to grain consumption than whole grain foods among most ethnic/racial groups. Grain foods made an important contribution to the daily intakes of thiamin, riboflavin, niacin, vitamin B6, and folic acid. This study provides much needed quantified evidence regarding the degree to which grain foods contribute to ethnic-specific diets, which can be used for the development of health strategies and ethnic-specific dietary guidance. These descriptive data will be valuable for future etiological studies conducted on the role of B vitamins on health and chronic disease, especially among high-risk populations.

## Competing interests

The authors have no conflict of interest to report.

## Authors’ contributions

This research was conceptualized by Sangita Sharma and Laurence Kolonel. Sangita Sharma and Tony Sheehy drafted the original manuscript, and all authors provided critical review and final approval of the manuscript.
